# Bone marrow-derived cells for cardiovascular cell therapy: an optimized GMP method based on low-density gradient improves cell purity and function

**DOI:** 10.1186/s12967-014-0276-0

**Published:** 2014-09-27

**Authors:** Marina Radrizzani, Viviana Lo Cicero, Sabrina Soncin, Sara Bolis, Daniel Sürder, Tiziano Torre, Francesco Siclari, Tiziano Moccetti, Giuseppe Vassalli, Lucia Turchetto

**Affiliations:** Cell Therapy Unit, Cardiocentro Ticino, Via Tesserete 48, 6900 Lugano, Switzerland; Division of Cardiology, Cardiocentro Ticino, Lugano, Switzerland; Division of Cardiac Surgery, Cardiocentro Ticino, Lugano, Switzerland

**Keywords:** Cell therapy, Bone marrow cells, Stem cells, Good manufacturing practices, Cardiovascular diseases

## Abstract

**Background:**

Cardiovascular cell therapy represents a promising field, with several approaches currently being tested. The advanced therapy medicinal product (ATMP) for the ongoing METHOD clinical study (“Bone marrow derived cell therapy in the stable phase of chronic ischemic heart disease”) consists of fresh mononuclear cells (MNC) isolated from autologous bone marrow (BM) through density gradient centrifugation on standard Ficoll-Paque. Cells are tested for safety (sterility, endotoxin), identity/potency (cell count, CD45/CD34/CD133, viability) and purity (contaminant granulocytes and platelets).

The aims of the present work were (1) to optimize the cell manufacturing process in order to reduce contaminants and (2) to implement additional assays in order to improve product characterization and evaluate product stability.

**Methods:**

BM-MNC were isolated by density gradient centrifugation on Ficoll-Paque. The following process parameters were optimized throughout the study: gradient medium density; gradient centrifugation speed and duration; washing conditions.

Differential cell count was performed by an automated hematology cell analyzer. Immunophenotype and cell viability were determined by flow cytometry. Functional hematopoietic and mesenchymal precursors and cells with angiogenic potential were assessed by colony-forming assays, cell invasion capacity by a fluorimetric assay. Sterility was tested using an automated microbial detection system, endotoxin by a kinetic chromogenic Limulus amebocyte lysate test. T-test was used for statistical analysis.

**Results:**

A new manufacturing method was set up, based on gradient centrifugation on low density Ficoll-Paque, followed by 2 washing steps, of which the second one at low speed. It led to significantly higher removal of contaminant granulocytes and platelets, improving product purity; the frequencies of CD34^+^ cells, CD133^+^ cells and functional hematopoietic and mesenchymal precursors were significantly increased.

The process was successfully validated according to Good Manufacturing Practices.

The resulting ATMP mainly consisted of viable MNC including CD34^+^ and CD133^+^ cell subsets (2.98% ± 1.90% and 0.83% ± 1.32%, respectively), CD184/CXCR4^+^ cells (34% ± 15%), CD34^+^/CD133^+^/CD309^+^ endothelial precursors (44 ± 21 in 10^6^ total cells), cells with invasion capacity, functional hematopoietic and mesenchymal precursors, cells with angiogenic potential; it was stable for 20 hours at 10°C.

**Conclusions:**

The methodological optimization described here resulted in a significant improvement of ATMP quality, a crucial issue to clinical applications in cardiovascular cell therapy.

## Background

Cardiovascular cell therapy represents a promising field, with several approaches currently being tested for the treatment of both heart disease and peripheral vascular diseases [1-3]. According to current European regulations [4-6], cell-based products such as bone marrow (BM)-derived cells for cardiovascular applications are defined as advanced therapy medicinal products (ATMP) and must be prepared according to Good Manufacturing Practice (GMP) standards. In this context, the development and validation of properly designed cell manufacturing and testing methods [7,8] are of paramount importance for successful translational research. The manufacturing process has to be carefully defined and validated to ensure product consistency [7]. A suitable Quality Control (QC) strategy has to be designed for every specific ATMP, aiming at evaluating its safety, identity, purity and potency [7-9]. Safety testing should encompass sterility and lack of endotoxin, at least. The identity test panel includes cell morphology and immunophenotype. These tests also provide information on ATMP purity, as they detect undesirable impurities such as contaminating cell types. Potency is defined as a measure of biological activity. A potency assay should be based on a defined biological effect closely related to the mechanism(s) responsible for the functional benefits [7]. Cell viability is an important component of the potency of cell-based ATMP; however, additional parameters of biological activity should also be tested [10].

Release specifications (i.e. acceptance criteria to be met by a product lot in order to be administered to a patient) need to be defined for safety, which is generally evaluated by assays described in European Pharmacopoeia (EP) (compendial assays), and for crucial parameters such as cell viability. For other parameters, mostly evaluated by non-compendial assays developed on a product-specific basis, data may be recorded for information only, at least during the initial phases of clinical development.

Our Cell Therapy Unit, authorized since 2008 for the production of ATMP, is focused on development activities aimed at modifying research grade cell products to obtain high quality, clinical grade cell products.

The ATMP for the ongoing METHOD study (“Bone marrow derived cell therapy in the stable phase of chronic ischemic heart disease”) [11] (ClinicalTrials.gov Identifier: NCT01666132; initial feasibility phase: 8/10 patients treated) consists of fresh mononuclear cells (MNC) isolated from autologous BM by density gradient centrifugation; cells are formulated in 5% human serum albumin (HSA) and tested for safety (sterility, endotoxin), identity/potency (cell count, CD45/CD34/CD133, viability) and purity (evaluation of contaminant granulocytes (GRA) and platelets (PLT).

The aims of the present work were (1) to optimize the manufacturing process in order to reduce contaminants; and (2) to set-up additional identity and potency assays in order to improve product characterization and evaluate product stability.

These improvements were crucial to the upcoming second phase of the METHOD trial, as well as to planned clinical trials, such as the CIRCULATE study (“Bone Marrow Derived Cell Therapy in Peripheral Artery Disease”) in patients with critical limb ischemia. They have been explicitly requested by Swiss regulatory authorities (Swissmedic).

## Methods

### BM harvesting and MNC isolation

Iliac crest BM was harvested in the patients who had been enrolled in the clinical trials SWISS-AMI (ClinicalTrials.gov Identifier: NCT00355186) [12,13] and METHOD (ClinicalTrials.gov Identifier: NCT01666132) [11] (target BM volume: 50 mL and 110 mL, respectively). Due to limited availability of iliac crest BM samples, sternal BM (volume: 54 ± 20 mL; n = 71) collected during cardiac artery bypass grafting surgery was used for the majority of development experiments, as preliminary results demonstrated a close similarity of sternal and iliac crest BM with respect to most analytical parameters (data not shown). All patients signed informed consent for BM donation. To prevent clotting, 1 mL of a solution containing 1000 international units of heparin (Drossapharm AG, http://www.drossapharm.ch) was added to each 10 mL BM sample. The BM was filtered through a 100 μm cell strainer (BD Biosciences, http://www.bdbiosciences.com) and diluted 1:2 in Dulbecco Phosphate Buffered Saline without Ca^2+^ and Mg^2+^ (D-PBS) (LiStarFish, http://www.listarfish.it). MNC were isolated by density gradient centrifugation on Ficoll-Paque PREMIUM (GE Healthcare, http://www.gelifesciences.com), followed by washing in D-PBS; 5% HSA (CSL Behring AG, http://www.cslbehring.ch) was used as formulation medium, and filtration through a 70 μm cell strainer (BD Biosciences) was performed as final process step.

Several process parameters were optimized throughout the present study: gradient medium density; gradient centrifugation speed and duration; washing conditions. The following density gradient media were used: Ficoll-Paque PREMIUM 1.077 (density: 1.077 g/L), thereafter indicated as standard Ficoll-Paque, and Ficoll-Paque PREMIUM 1.073 (density: 1.073 g/L), thereafter indicated as low density Ficoll-Paque.

### Differential cell count

An automated hematology cell analyzer (ABX Micros 60, Horiba medical, http://www.horiba.com/medical) was used to determine total White Blood Cells (WBC), percentages of lymphocytes (LYM), monocytes (MON), GRA, PLT, and Hematocrit. MNC were calculated by adding up LYM and MON. The test was carried out according to EP [14].

GRA removal, PLT removal and MNC yield were calculated according to the following formulas:

$$ \%\ \mathrm{P}\mathrm{L}\mathrm{T}\ \mathrm{removal}=\frac{total\ pre\  manipulation\ PLT- total\  post\  manipulation\ PLT\ }{total\ pre\  manipulation\ PLT} \times 100 $$

$$ \%\ \mathrm{G}\mathrm{R}\mathrm{A}\ \mathrm{removal}=\frac{total\ pre\  manipulation\ GRA- total\  post\  manipulation\ GRA\ }{total\ pre\  manipulation\ GRA} \times 100 $$

$$ \%\ \mathrm{M}\mathrm{N}\mathrm{C}\ \mathrm{yield}=\frac{total\  post\  manipulation\ MNC}{total\ pre\  manipulation\ MNC} \times 100 $$

### Immunophenotype & cell viability

Immunophenotype (CD45 and CD34, Beckman Coulter, https://www.beckmancoulter.com; or Miltenyi Biotec, https://www.miltenyibiotec.com; CD133, Miltenyi Biotec; CD184/CXCR4: BD Biosciences) and cell viability (7-AAD, Beckman Coulter or PI, Miltenyi Biotec) were determined by flow cytometry (FC 500 System, Beckman Coulter or MACSQuant Analyzer, Miltenyi Biotec). The tests were run according to EP [14,15].

Endothelial progenitor cells (EPC) were determined using a commercial kit (“EPC enrichment and enumeration kit”, Miltenyi Biotec) specifically designed for the enumeration of circulating EPC from peripheral blood, cord blood, leukapheresis products, or EPC from BM, based on the expression of CD34, CD133 and CD309 (VEGFR-2/KDR).

### Colony-forming cell (CFC) assay

For the enumeration of hematopoietic stem cell precursors, cells were suspended in Methocult® H4034 (StemCell Technologies, http://www.stemcell.com), then seeded in 35 mm dishes (1–2 × 10^4^ viable cells/dish) and incubated at 37°C, 5% CO_2_. After 14 days, plates were microscopically scored for the presence of colonies. The assay was carried out according to EP [16].

### Colony-forming unit-fibroblast (CFU-F) assay

For the enumeration of mesenchymal stem cell precursors, cells were suspended in Mesencult®-XF (StemCell Technologies), then seeded in 100 mm dishes (5 × 10^5^, 1 × 10^6^, 2 × 10^6^ viable cells/dish) and incubated at 37°C, 5% CO_2_. After 14 days, plates were fixed with methanol (International VWR, https://ch.vwr.com), stained with Giemsa solution (Merck, http://www.merck.com) and scored for the presence of colonies.

### Colony-forming unit-endothelial cell (CFU-EC) assay

For the enumeration of angiogenic precursors, cells were suspended in Complete CFU-Hill medium (StemCell Technologies), then seeded in fibronectin-coated 6-well plates (BD Biosciences) at 5 × 10^6^ viable cells/well, and incubated at 37°C, 5% CO_2_. After 2 days, non-adherent cells were collected, transferred onto fibronectin-coated 24-well plates (10^6^ viable cells/well) and incubated for 5 days. The wells were then fixed with methanol (International VWR), stained with Giemsa solution (Merck) and scored for the presence of colonies.

### Invasion assay

Cells were labelled with BD™ DilC_12_(3) (BD Biosciences), suspended in X-Vivo 10 (Lonza, http://www.lonza.com), then seeded (10^6^ cells/w) in BD Biocoat™ Matrigel™ invasion chambers as well as control inserts (BD Biosciences) in 24 well plates; complete CFU-Hill liquid medium (StemCell Technologies) was added to the wells, below the inserts. After 24 hours at 37°C, 5% CO_2_, the fluorescence of invading cells was determined by a bottom fluorescence plate reader (Infinite F200, Tecan, http://www.tecan.com). The Invasion Index was calculated as follows:

$$ \frac{\mathrm{mean}\ \mathrm{Relative}\ \mathrm{Fluorescence}\ \mathrm{Units}\ \mathrm{of}\ \mathrm{cells}\ \mathrm{invading}\ \mathrm{through}\ \mathrm{Matrigel}}{\mathrm{mean}\ \mathrm{Relative}\ \mathrm{Fluorescence}\ \mathrm{Units}\ \mathrm{of}\ \mathrm{cells}\ \mathrm{invading}\ \mathrm{through}\ \mathrm{control}\ \mathrm{inserts}} \times 100 $$

### Sterility

The test was carried out according to EP [17] using an automated microbial detection system (BacT/ALERT® 3D, bioMérieux, http://www.biomerieux.com). Results were released not less than 7 days after sample inoculum.

### Bacterial endotoxins

This test was carried out according to EP [18] with a chromogenic technique using a synthetic peptide-chromogenic substrate complex cleaved by the reaction of endotoxins with Limulus amebocyte lysate. The endosafe®-PTS™ system (Charles River, http://www.criver.com) was used.

### Statistical analysis

Statistical analysis was performed with the two-tailed unpaired or paired t-test (Microsoft Excel software), as indicated in the text.

## Results

The BM-MNC manufacturing method currently used in the ongoing feasibility phase of the METHOD trial [11] is based on density gradient centrifugation (719 × g, 20’ at 20°C) on standard Ficoll-Paque (1.077 g/L), followed by 3 washing steps (582 × g, 10’ at 4°C).

The resulting product is composed of WBC with morphological characteristics of LYM (mean value ± standard deviation (SD)), 53% ± 16%), MON (15% ± 4%), and GRA (32% ± 18%) (n = 8). The PLT/WBC ratio is 4 ± 2. The MNC fraction comprising LYM and MON is considered the active fraction, whereas GRA and PLT represent impurities. For all 8 clinical lots produced so far, the levels of impurities were in compliance with product release specifications (GRA ≤ 75%, PLT/WBC ≤ 40; see below). Nonetheless, minimization of such impurities is desirable. Thus, development experiments were initiated to optimize the BM-MNC manufacturing process.

Preliminary tests performed on a limited number of samples indicated that gradient centrifugation parameters (relative centrifugation force and duration) may not affect process performance in terms of impurity clearance or cell yield (Table 1); however, changing the gradient medium may improve GRA removal (Table 2), while adding a low-speed washing step may improve PLT removal efficiency, with no negative impact on product potency (Table 3).

**Table 1:** **Preliminary experiments: gradient centrifugation parameters do not affect impurities’ removal nor cell yield (n = 5)**

**Gradient medium**	**Centrifugation parameters**	**% PLT removal**	**% GRA removal**	**% MNC yield**
Standard Ficoll-Paque (1.077 g/L)	719 × g, 20°C, 20’	93.73 ± 1.63	96.02 ± 3.05	24.15 ± 22.90
	400 × g, 20°C, 30’	92.05 ± 1.36	93.41 ± 1.64	27.41 ± 15.98

Five sternal bone marrow samples were processed in parallel with the same gradient medium but using different centrifugation parameters, as indicated. Data are reported as mean ± standard deviation. No statistically significant differences were detected (p > 0.05, paired T-test).

*PLT*: Platelets; *GRA*: Granulocytes; *MNC*: Mononuclear cells (lymphocytes + monocytes).

**Table 2:** **Preliminary experiments: using a low density gradient medium ameliorates granulocytes’ removal (n = 5)**

**Centrifugation parameters**	**Gradient medium**	**% PLT removal**	**% GRA removal**	**% MNC yield**
400 × g, 20°C, 30’	Standard Ficoll-Paque (1.077 g/L)	91.49 ± 7.14	94.55 ± 3.75	21.03 ± 16.14
	Low Density Ficoll-Paque (1.073 g/L)	91.84 ± 7.82	97.28 ± 2.12**	12.12 ± 9.45*

Five sternal bone marrow samples were processed in parallel using the same centrifugation parameters but different gradient media, as indicated. Data are reported as mean ± standard deviation. *p < 0.05; **p < 0.01, paired T-test.

*PLT*: Platelets; *GRA*: Granulocytes; *MNC*: Mononuclear cells (lymphocytes + monocytes).

**Table 3:** **Preliminary experiments: low speed washing improves platelets’ removal (n = 14)**

**Washing speed**	**% PLT removal**	**% MNC yield**	**CFC/10** ^**6**^ **cells**	**CFU-F/10** ^**6**^ **cells**	**Invasion index**
400 × g	91.59 ± 8.74	13.43 ± 9.26	11 310 ± 2 847	18 ± 10	37 ± 14
100 × g	95.11 ± 4.12**	10.69 ± 8.29**	12 620 ± 2 581*	18 ± 10	47 ± 7

Mononuclear cells were isolated from 14 bone marrow samples through density gradient on standard or low density Ficoll-Paque, then washed in Dulbecco’s phosphate buffered saline without Ca^2+^ and Mg^2+^; for the last washing step, each sample was divided in 2 aliquots that were centrifuged in parallel at 400 × g and 100 × g, as indicated.

*PLT*: Platelets; *MNC*: Mononuclear cells (lymphocytes + monocytes); *CFC*: Colony Forming Cells; *CFU-F*: Colony Forming Units-Fibroblast.

Data are reported as mean ± standard deviation. *p < 0.05; **p < 0.01, paired T-test.

CFC assay was performed on 8 product batches only, CFU-F and Invasion assays on 4 product batches only.

Based on such initial results, a new manufacturing method was designed, relying on the use of low-density Ficoll-Paque (1.073 g/L) as the gradient medium. The gradient step (400 × g, 30’ at 20°C) was followed by 2 washing steps at 20°C, of which the second one was performed at low speed (washing #1: 400 × g; #2: 100 × g) in order to reduce the possibility that PLT may sediment together with MNC and be recovered in the cell pellet.

The new manufacturing method was compared with the previous one in terms of overall efficiency (Table 4). It resulted in significantly improved removal of contaminant PLT and GRA (p < 0.01). On the other hand, the MNC yield was diminished.

**Table 4:** **New manufacturing method overall efficiency: improved contaminants’ removal**

	**% PLT removal**	**% GRA removal**	**% MNC yield**
Current method (n = 43)^a^	89 ± 7	91 ± 4	25 ± 11
New method (n = 24)^b^	97 ± 1**	95 ± 3**	13 ± 7**

Data are reported as mean ± standard deviation.

*PLT*: Platelets; *GRA*: Granulocytes; *MNC*: Mononuclear cells (lymphocytes + monocytes).

^a^iliac crest samples (n = 33) + sternal samples (n = 10); ^b^sternal samples only; **p < 0.01 (unpaired T-test).

### Product purity

The new processing method resulted in improved product purity (Table 5): MNC were significantly increased (p < 0.01), mainly due to an increased MON fraction, whereas contaminant GRA were significantly reduced (p < 0.01). The mean PLT/WBC ratio was not significantly affected (p > 0.05); however, its variability was drastically reduced.

**Table 5:** **New manufacturing method: improved product purity**

	**% Lymphocytes**	**% Monocytes**	**% MNC**	**% Granulocytes**	**PLT/WBC**
Current Method (n = 43)^a^	48 ± 13 (21–71)	9 ± 3 (5–15)	57 ± 15 (26–84)	43 ± 14 (16–74)	6 ± 5 (1–28)
New Method (n = 24)^b^	52 ± 11 (34–69)	15 ± 4 ** (8–22)	66 ± 11** (42–85)	34 ± 11** (15–58)	5 ± 2 (2–10)

Data are reported as mean ± standard deviation (range).

*MNC*: Mononuclear cells (lymphocytes + monocytes); *PLT*: Platelets; *WBC*: White Blood Cells.

^a^iliac crest (n = 33) and sternal (n = 10) bone marrow samples; ^b^sternal samples only; **p < 0.01 (unpaired T-test).

Such results allowed us to update product release specifications for crucial identity/purity parameters, as summarized in Table 6. The new specifications were set on the basis of results observed during process development (reported in Table 5, bottom line). The highest acceptable values of LYM and MON were defined considering the corresponding experimental mean values minus 2 SD. The highest acceptable values of GRA and PLT/WBC were defined based on the corresponding experimental mean values plus 2 SD.

**Table 6:** **New specifications for product release**

	**% Lymphocytes**	**% Monocytes**	**% Granulocytes**	**PLT/WBC**
Current specification	≥ 25	≥ 4	≤ 75	≤ 40
Updated specification	≥ 30	≥ 8	≤ 55	≤ 10

*PLT*: Platelets; *WBC*: White Blood Cells.

All the characterization results obtained on product lots manufactured during process development according to the new method (Table 7; n = 20) were in compliance with the new specifications.

**Table 7:** **Product characterization after optimization of cell processing**

**Parameter**	**Spec.**	**Mean**	**SD**	**n**	**Min**	**Max**
Cell concentration (WBC/ml)	FIO	1.60 × 10^7^	1.04 × 10^7^	20	4.40 × 10^6^	4.39 × 10^7^
Lymphocytes (%)	≥ 30	62	12	20	41	83
Monocytes (%)	≥ 8	14	4	20	8	23
Granulocytes (%)	≤ 55	24	10	20	6	49
Platelet concentration (PLT/ml)	FIO	2.66 × 10^7^	2.42 × 10^7^	20	2.50 × 10^6^	9.60 × 10^7^
PLT/WBC	≤ 10	1.75	0.90	20	0.39	3.84
RBC concentration (RBC/ml)	FIO	2.23 × 10^7^	1.32 × 10^7^	16	1.00 × 10^7^	6.30 × 10^7^
RBC/WBC	FIO	1.67	0.41	16	0.77	2.27
Hematocrit (%)	≤ 3	0.29	0.18	15	0.10	0.85
CD45^+^/CD34^+^ (%)	FIO	2.98	1.90	18	0.80	7.74
CD45^+^/CD133^+^ (%)	FIO	0.83	1.32	18	0.02	4.26
CD133^+^ among CD34^+^ cells (%)	FIO	53	10	6	40	68
CD45^+^/CD184^+^ (%)	FIO	34	15	9	10	57
EPC/10^6^ WBC (CD34^+^/CD133^+^/CD309^+^)	FIO	44	21	5	12	60
Cell viability (%)	≥ 70	90	5	18	79	98
CFC (colonies/10^6^ cells)	FIO	6 998	4 363	7	1 417	14 400
CFU-F (colonies/10^6^ cells)	FIO	56	40	7	11	131
CFU-EC (colonies/10^6^ cells)	FIO	31	19	12	6	61
Invasion (invasion index)	FIO	47	18	12	25	85

*WBC*: White Blood Cells; *PLT*: Platelets; *RBC*: Red Blood Cells; *EPC*: Endothelial Progenitor Cells; *CFC*: Colony Forming Cells; *CFU-F*: Colony Forming Units-Fibroblasts; *CFU-EC*: Colony Forming Units-Endothelial Cells; *FIO*: For information only.

### Immunophenotype

**Figure 1:**
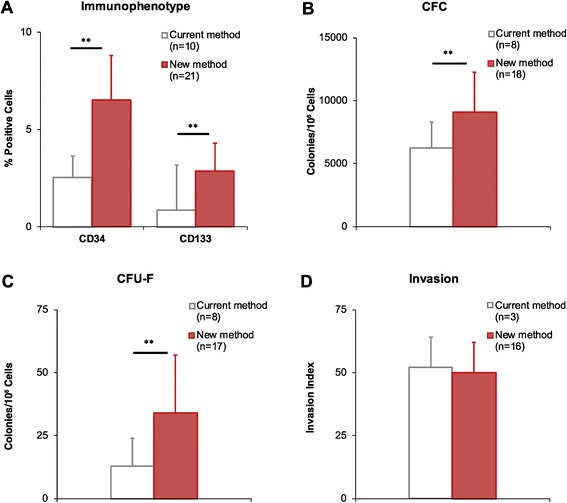
**New manufacturing method: improved product immunophenotype and potency.** Sternal bone marrow samples were processed according to the current or the new manufacturing method, as indicated, then tested as follows: **(A)** Immunophenotype, to detect CD34^+^ and CD133^+^ stem cells. **(B)** Colony Forming Cell assay (CFC), to detect hematopoietic precursor cells. **(C)** Colony Forming Unit-Fibroblast assay (CFU-F), to detect mesenchymal precursor cells. **(D)** Invasion assay, to detect cells with invasion capacity. Data are expressed as mean values; error bars represent standard deviations; *p < 0.05; **p < 0.01, unpaired T-test.

The new manufacturing method led to significantly higher percentages (p < 0.01) of CD34 and CD133 expressing cells (Figure 1A), accounting for 2.98% ± 1.90% and 0.83% ± 1.32% of cells, respectively, in the new ATMP (Table 7; n = 18). Among CD34^+^ cells, 53% ± 10% were CD133^+^ (n = 8), in agreement with data reported for adult BM [19]. The product also contained a consistent fraction (34% ± 15%; n = 18) of cells expressing CD184/CXCR4, the receptor for stromal cell-derived factor-1 (SDF-1), a functional marker of BM-MNC [20]. CD34^+^/CD133^+^/CD309^+^ EPC (Table 7) accounted for 0.004% of WBC (44 ± 21 cells in 10^6^ total cells). Release specifications were not defined for the immunophenotype-specific parameters; data are collected for information only.

### Product potency

The product potency was evaluated by assessing cell viability, invasion capacity and presence of functional hematopoietic precursors, mesenchymal precursors, and cells with angiogenic potential using CFC, CFU-F, and CFU-EC assays, respectively. The new manufacturing method significantly improved product potency in terms of functional hematopoietic (CFC, Figure 1B; p < 0.01) and mesenchymal precursors (CFU-F, Figure 1C; p < 0.01), but not invasion capacity (Figure 1D; p > 0.05).

The new ATMP (Table 7) was characterized by high cell viability (90% ± 5%), well above the 70% specification, which is considered the minimum acceptable value for cell therapy products [21].

It contained hematopoietic progenitor cells (CFC, 6 998 ± 4 363 colonies/10^6^ cells), mesenchymal progenitor cells (CFU-F, 56 ± 40 colonies/10^6^ cells), cells with angiogenic potential (CFU-EC, 31 ± 19 colonies/10^6^ cells) and cells with invasion capacity (invasion index 47% ± 18%); no specifications were defined for such parameters.

### GMP validation

The new manufacturing method was validated by performing three production runs and testing the resulting BM-MNC preparations as summarized in Table 8. Three independent BM samples (100 ± 26 mL) were processed according to GMP in the classified area dedicated to sterile manufacturing, giving rise to 3 BM-MNC lots (18.5 ± 0.1 mL) that were tested in the GMP QC area. Defined specifications were in place for safety (sterility, bacterial endotoxins), identity/purity (LYM, MON, GRA, PLT/WBC, Hematocrit), and cell viability. The corresponding analytical methods were validated according to GMP for appropriate characteristics including specificity, repeatability, and intermediate precision [22,23]. For the three BM-MNC lots, results of all of these tests complied with specifications (Table 8).

**Table 8:** **GMP process validation**

**Parameter**	**Specification**	**Results – Lot Number:**		
		**002.12**	**004.12**	**005.12**
Visual control: appearance	Limpid; absence of micro aggregates, colorless to slightly hematic	Limpid, absence of micro aggregates, colorless	Limpid, absence of micro aggregates, colorless	Limpid, absence of micro aggregates, colorless
Integrity of primary container	Intact	Intact	Intact	Intact
Sterility	No growth	No growth	No growth	No growth
Bacterial endotoxins (EU/ml)	< 5.00	< 5.00	< 5.00	< 5.00
Cell concentration (WBC/ml)	FIO	1.20 × 10^6^	2.40 × 10^6^	1.25 × 10^6^
Lymphocytes (%)	≥ 30	71	57	61
Monocytes (%)	≥ 8	14	16	14
Granulocytes (%)	≤ 55	15	27	25
Platelet concentration (PLT/ml)	FIO	6.30 × 10^6^	1.50 × 10^7^	7.50 × 10^6^
PLT/WBC	≤ 10	5	6	6
Hematocrit (%)	≤ 3	0.1	0.1	0.05
CD45^+^/CD34^+^ (%)	FIO	2.94	8.41	8.95
CD45^+^/CD133^+^ (%)	FIO	1.42	3.8	4.25
Cell viability (%)	≥ 70	74	84	99
CFC (colonies/10^6^ cells)	FIO	2 450	9 850	11 550
CFU-F (colonies/10^6^ cells)	FIO	44	55	36
Invasion (invasion index)	FIO	35	59	47

*EU*: Endotoxin Units; *WBC*: White Blood Cells; *PLT*: Platelets; *CFC*: Colony Forming Cells; *CFU-F*: Colony Forming Units-Fibroblasts; *FIO*: For information only.

The process guaranteed product sterility, absence of bacterial endotoxins, consistent appearance, integrity of the primary containers, and consistent product purity. GRA (15-27%) were well below the new highest acceptable value (55%) and the PLT/WBC ratio (5–6) was within the new specification (≤10). The product mainly consisted of MNC (73-85%) and contained cells expressing CD34 (2.94-8.95%) and CD133 (1.42-4.25%). Cell viability was 74-99%. In terms of potency, the product showed detectable functional hematopoietic (CFC) and mesenchymal (CFU-F) precursors as well as cells with invasion capacity.

In summary, validation results were consistent with those obtained during product development and characterization. Accordingly, we considered that the new manufacturing process was successfully validated for safety, purity, identity and potency aspects.

### Stability study

**Figure 2:**
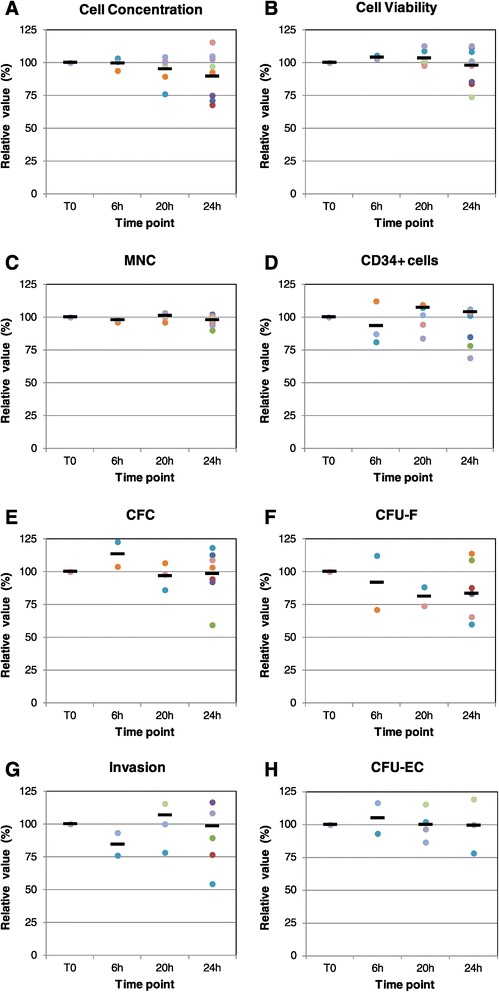
**Product stability.** Nine sternal bone marrow samples were processed according to the new manufacturing method. The resulting product batches entered a stability program: they were stored at 10°C, and tested at 0-6-20-24 hours, for the following parameters: **(A)** Cell concentration. **(B)** Cell viability. **(C)** Percentage of mononuclear cells (MNC) (lymphocytes + monocytes). **(D)** Percentage of CD34^+^ cells. **(E)** Colony Forming Cell assay (CFC), to detect hematopoietic precursor cells. **(F)** Colony Forming Unit-Fibroblast assay (CFU-F), to detect mesenchymal precursor cells. **(G)** Invasion assay, to detect cells with invasion capacity. **(H)** Colony Forming Unit-Endothelial Cell assay (CFU-EC), to detect cells with angiogenic potential Data are expressed as relative values in respect to time 0. Each dot represents a single batch (identified by a distinct color), while black dashes indicate mean values. $\mathrm{Relative}\ \mathrm{value}\ \left(\%\right)=\frac{\mathrm{value}\ \mathrm{at}\ \mathrm{the}\ \mathrm{indicated}\ \mathrm{time}\ \mathrm{point}-\mathrm{value}\ \mathrm{at}\ \mathrm{time}\ 0}{\mathrm{value}\ \mathrm{at}\ \mathrm{time}\ 0}\mathrm{x}\ 100$.

In order to define product shelf-life, a real-time stability study was performed at 10°C, previously identified in our lab as the optimal storage temperature for fresh BM-MNC (data not shown). A practical approach was used: stability specifications corresponded to release specifications (sterility: absence of bacterial growth, endotoxin ≤ 5.0 EU/ml, cell viability ≥ 70%, LYM ≥ 30%, MON ≥ 8%, GRA ≤ 75%, hematocrit ≤ 3.0). The stability study was also extended to functional parameters not yet subjected to release specifications. As additional stability criteria, the maximum decrease of each parameter relative to the corresponding baseline value was set at 25% (i.e. relative value ≥ 75%, see Figure 2). All the cell batches tested were in compliance with stability specifications for at least 20 hours (data not shown). For all parameters, differences between results obtained at baseline, 20 hours, and 24 hours were not significant. Relative values remained ≥75% of baseline until 20 hours for cell concentration (Figure 2A), viability (Figure 2B), MNC (Figure 2C), CD34^+^ cells (Figure 2D), CFC (Figure 2E), invasion (Figure 2G) and CFU-EC (Figure 2H), whereas some CFU-F values were between 50 and 75% (Figure 2F). According to these results, the product shelf life was set at 20 hours.

## Discussion

This work focused on the development and validation of a novel GMP manufacturing and testing strategy for BM-MNC as ATMP for cardiovascular cell therapy. Our goals were (1) to optimize the process in order to reduce contaminant GRA and PLT, and (2) to set-up additional identity and potency assays in order to better characterize the cell product and evaluate its stability.

In our product, the MNC fraction comprising LYM and MON is considered the active component, whereas PLT and GRA represent impurities. The role of PLT within a cell therapy product is controversial, presumably due to their complex biology: they may exert potential beneficial effects on cell engraftment and tissue regeneration [24], but also negative effects. For example, in the context of the REPAIR-AMI trial, the number of contaminating PLT in the BM-MNC product was reported to inversely correlate with LVEF recovery in patients after cell therapy, even though this negative effect of PLT was less pronounced than that of contaminant RBC; on the contrary, contaminant neutrophils had no significant impact [25]. Regardless of the therapeutic effect, BM-MNC purity is an important parameter with respect to the interpretation of the results of clinical trials. Increasing the product purity has been also specifically requested by regulatory authorities. The majority of development experiments were performed using sternal BM samples, harvested from patients undergoing cardiac surgery involving sternotomy. Such samples, showing a close similarity to iliac crest samples with respect to most analytical parameters, were more easily available in our clinical context with respect to iliac crest BM.

The previous manufacturing protocol used in the initial phase of the METHOD study [11] and in the “Swiss multicenter intracoronary stem cells study in acute myocardial infarction clinical trial” (SWISS-AMI) [12,13] was derived from prior studies [26] with minor modifications. The new BM-MNC manufacturing protocol relies on the use of a low-density variant of Ficoll-Paque, i. e. Ficoll-Paque PREMIUM 1.073, which was selected based on previous evidence that BM-MNC isolated by Ficoll-Paque with different densities may differ in composition [27]. In agreement with Grisendi et al. [27], we observed that low-density Ficoll-Paque leads to cells with higher mesenchymal stem cell clonogenic potential compared to standard Ficoll-Paque (Figure 1C). However, those authors reported a similar recovery of total nucleated cells for the two media, whereas in our hands the MNC yield was significantly reduced using the new protocol (Table 4), presumably due to the effect of low-density Ficoll-Paque (Table 2). Nevertheless, this limitation is offset by several advantages including higher purity (Table 5) and higher content of hematopoietic and mesenchymal precursors in cell products manufactured with the new method (Figure 1).

Unlike Grisendi et al. [27], we evaluated additional methodological aspects besides the gradient medium. Gradient centrifugation parameters were adjusted (from 719 × g, 20’ at 20°C to 400 × g, 30’ at 20°C) to better adhere to the Ficoll-Paque manufacturer’s instructions; such adjustment itself had no impact on overall process performance (Table 1).

The washing procedure was also modified (from three washing steps at 582 × g, 10’ at 4°C, to two washing steps at 400 × g and 100 × g, respectively, both at 20°C), on the basis of Ficoll-Paque manufacturer’s indications suggesting two centrifugations, one of which may be performed at low speed to improve PLT removal. In our hands, the use of a low-speed centrifugation step indeed contributed to PLT abatement, even though it further reduced MNC yield (Table 3). The filtration steps were not optimized as they do not affect process performance (data not shown). The purpose of the initial filtration (100 μm) is to remove cell clumps or BM tissue fragments that could interfere with subsequent process steps, while the goal of the final filtration (70 μm) is to withdraw possible cell aggregates from the final product. Overall, the new method significantly ameliorated product quality.

With respect to the QC strategy applied in most previous BM-MNC cardiovascular cell therapy studies [2,28,29], our approach is more stringent in terms of safety, as in those studies cells were tested for sterility/microbiological contamination [30-34] but not for endotoxin. Endotoxin testing is important because high endotoxin levels as a result of bacterial contamination and/or improper quality of manufacturing materials and reagents cannot be ruled out *a priori*. Moreover, this is the only safety test which yields results before fresh product infusion, since microbiological assays take 7 days or longer. The specification for endotoxin (<5 Endotoxin Units/mL) has been set according to EP [35].

Cell viability was evaluated in most trials [26,30,32,34,36-44] and defined release criteria were reported by some authors, e.g. > 70% viable cells in the FOCUS-CCTRN trial [30,31] and > 80% in the REPAIR-AMI trial [31]. In our case, the specification was set at ≥ 70% in accordance to recent FDA guidelines [21].

Cell immunophenotype (mainly CD45 and CD34) was analyzed in most previous studies [30-34,36,39,41-44], even though no release specifications were reported. We extended the immunophenotypic analysis to include not only CD133, a stem cell marker expressed by a subset of CD34^+^ cells [19,45] but also CD184/CXCR4 (SDF-1 receptor), a marker of functional BM-MNC activity. Seeger et al. [20] reported that injection of CXCR4^+^ BM-MNC in mice with hindlimb ischemia significantly improved the recovery of perfusion compared to injection of CXCR4-negative BM-MNC; likewise, capillary density was significantly increased in mice treated with CXCR4^+^ BM-MNC. The higher migratory capacity of such cells and the release of paracrine factors may have contributed to tissue repair [20]. A correlation between in vitro cell potency and long term clinical outcome has been recently shown for BM-MNC therapy of acute myocardial infarction [46].

In the present study, the presence of CD34^+^ CD133^+^ CD309^+^ EPC [47] was evaluated on representative BM-MNC batches (Table 7, n = 5); however, it is not part of the QC release panel due to the limited number of available cells in clinical batches and the relatively high cell number required for this analysis.

In terms of functional cell characterization, we included in QC release panel colony-forming assays for both hematopoietic (CFC) and mesenchymal precursors (CFU-F), along with testing of invasion capacity, which were assessed in the REPAIR-AMI study [25] and, in part, in the PROVASA study [39]. In addition, we performed the CFU-EC assay to detect “angiogenic cells” [48].

In previous studies, limited data on cell characteristics may have hampered the assessment of critical cell product parameters. The poor product characterization also limited the evaluation of product comparability among cardiovascular cell therapy studies, thus contributing to variable clinical results [28]. It is well known that BM-MNC isolated through density gradient consist of a mixture of various cell subsets with different phenotype and function [49,50], including progenitor/stem cells such as hematopoietic stem cells, mesenchymal stem cells, EPC, multipotent adult mesenchymal progenitors and embryonic-like stem cells. The cell subsets responsible for beneficial effects of total BM-MNC in several clinical studies are not yet identified [20,51]. It is also unknown whether selected BM-derived cell subpopulations may be superior to unfractionated BM populations containing a mixture of differentiated and less differentiated cells, potentially enhancing their effect by cross-talking with each other [51].

Based on these considerations, our immunophenotypic and functional analysis of clinical grade BM-MNC may well contribute to the identification of product characteristics having impact on the clinical outcome in different patient populations, thus facilitating the identification of product critical quality attributes and the definition of release specifications for further clinical studies [52].

The last part of our work focused on product stability, an issue particularly relevant in the case when a freshly-derived cell product is delivered to the patient. Due to the peculiar nature of the product (live cells, intrinsic inter-batch variability due to differences among patients, limited product availability), the stability study was performed at the storage temperature only (10 ± 5°C), as accelerated and stressed conditions recommended in general stability guidelines [53,54] were considered not applicable for viable cells. Both release parameters and characterization parameters not yet subjected to release specifications were evaluated within the context of stability testing (Figure 2). Overall, the results suggested that the product shelf-life could be set at 20 hours. This time frame abundantly covers the time necessary for the release of the product: pre-infusion testing (endotoxin, cell concentration, cell viability, immunophenotype) generally requires approximately 2 hours, or 4 hours in case of retesting due to deviations or non-conformities; the remaining 16 hours may cover the transport to the clinical site and/or infusion delay due to clinical issues.

A limitation of the present study is the lack of *in vivo* experiments to demonstrate the superiority of BM-MNC manufactured according to the new protocol in terms of therapeutic potential. It could be interesting to address this issue in future pre-clinical studies, in animal models of hind limb ischemia or myocardial infarction.

Principles detailed in Swiss and European regulations for ATMP [4-6], as well as in the applicable European Medicinal Agency guidelines [7] and GMP guidelines [55], were taken into consideration throughout our development work. This approach allowed us to fulfil requests formulated by the competent regulatory authorities in view of the upcoming second phase of the METHOD trial [11] and of the new CIRCULATE study. Based on the results summarized here, included in the quality section of Investigational Medicinal Product Dossier, the CIRCULATE clinical trial was successfully submitted and recently got authorization.

## Conclusions

Methods for BM-MNC production and testing have been optimized and validated according to GMP. In particular, the manufacturing process has been redesigned, resulting in higher product purity and activity. Additional identity and potency assays have been set up in order to extend product characterization and evaluate product stability: an extended QC panel has been established, encompassing safety, identity/purity, and potency. Release specifications have been updated and product shelf-life has been defined based on experimental results obtained during development and GMP validation. The present work represents an example of constructive cooperation between a cell therapy manufacturing site and regulatory authorities, whose valuable inputs have been considered during product development.

## Abbreviations

ATMP: Advanced therapy medicinal product
BM: Bone marrow
CFC: Colony-forming cell
CFU-EC: Colony-forming unit-endothelial cell
CFU-F: Colony-forming unit-fibroblast
D-PBS: Dulbecco’s phosphate buffered saline without Ca^2+^ and Mg^2+^
EP: European Pharmacopoeia
EPC: Endothelial progenitor cells
GMP: Good Manufacturing Practice
GRA: Granulocytes
HSA: Human serum albumin
LYM: Lymphocytes
MNC: Mononuclear cells
MON: Monocytes
PLT: Platelets
QC: Quality control
SD: Standard deviations
SDF-1: Stromal cell-derived factor-1
WBC: White blood cells

## References

[1] Blum A, Balkan W, Hare JM (2012). Advances in cell-based therapy for peripheral vascular disease. Atherosclerosis.

[2] Raval Z, Losordo DW (2013). Cell therapy of peripheral arterial disease: from experimental findings to clinical trials. Circ Res.

[3] Schulman IH, Hare JM (2012). Key developments in stem cell therapy in cardiology. Regen Med.

[4] **Federal act on the transplantation of organs, tissues and cells - Swiss Confederation.** 2004.

[5] **Federal ordinance on the transplantation of human organs, tissues and cells - Swiss Confederation.** 2007.

[6] **Regulation (EC) No 1394/2007 of the European Parliament and of the Council of 13 November 2007 on advanced therapy medicinal products and amending Directive 2001/83/EC and Regulation (EC) No 726/2004.**

[7] **Guideline on Human Cell-based Medicinal Products, EMEA/CHMP/410869/2006.**.

[8] Rayment EA, Williams DJ (2010). Concise review: mind the gap: challenges in characterizing and quantifying cell- and tissue-based therapies for clinical translation. Stem Cells.

[9] **ICH Q6B “Specifications: Test Procedures and Acceptance Criteria for Biotechnological/Biological Products”.** 1999.

[10] **Guideline on potency testing of cell based immunotherapy medicinal products for the treatment of cancer, EMEA/CHMP/410869/2006.**

[11] Surder D, Radrizzani M, Turchetto L, Cicero VL, Soncin S, Muzzarelli S, Auricchio A, Moccetti T (2013). Combined delivery of bone marrow-derived mononuclear cells in chronic ischemic heart disease: rationale and study design. Clin Cardiol.

[12] Surder D, Schwitter J, Moccetti T, Astori G, Rufibach K, Plein S, Lo Cicero V, Soncin S, Windecker S, Moschovitis A, Wahl A, Erne P, Jamshidi P, Auf der Maur C, Manka R, Soldati G, Bühler I, Wyss C, Landmesser U, Lüscher TF, Corti R (2010). Cell-based therapy for myocardial repair in patients with acute myocardial infarction: rationale and study design of the SWiss multicenter Intracoronary Stem cells Study in Acute Myocardial Infarction (SWISS-AMI). Am Heart J.

[13] Surder D, Manka R, Lo Cicero V, Moccetti T, Rufibach K, Soncin S, Turchetto L, Radrizzani M, Astori G, Schwitter J, Erne P, Zuber M, Auf der Maur C, Jamshidi P, Gaemperli O, Windecker S, Moschovitis A, Wahl A, Bühler I, Wyss C, Kozerke S, Landmesser U, Lüscher TF, Corti R (2013). Intracoronary injection of bone marrow-derived mononuclear cells early or late after acute myocardial infarction: effects on global left ventricular function. Circulation.

[14] *European Pharmacopoeia 7.0, Section 2.7.29 (Nucleated cell count and viability).* Strasbourg, FR: European Directorate for the Quality of Medicines & HealthCare; 2010.

[15] *European Pharmacopoeia 7.0, Section 2.7.24 (Flow Cytometry).* Strasbourg, FR: European Directorate for the Quality of Medicines & HealthCare; 2010.

[16] *European Pharmacopoeia 7.0, Section 2.7.28 (Colony-forming cell assay for human hematopoietic progenitor cells).* Strasbourg, FR: European Directorate for the Quality of Medicines & HealthCare; 2010.

[17] *European Pharmacopoeia 7.0, Section 2.6.27 (Microbiological control for cellular products).* Strasbourg, FR: European Directorate for the Quality of Medicines & HealthCare; 2010.

[18] *European Pharmacopoeia 7.0, Section 2.6.14 (Bacterial endotoxins).* Strasbourg, FR: European Directorate for the Quality of Medicines & HealthCare; 2010.

[19] Yin AH, Miraglia S, Zanjani ED, Almeida-Porada G, Ogawa M, Leary AG, Olweus J, Kearney J, Buck DW (1997). AC133, a novel marker for human hematopoietic stem and progenitor cells. Blood.

[20] Seeger FH, Rasper T, Koyanagi M, Fox H, Zeiher AM, Dimmeler S (2009). CXCR4 expression determines functional activity of bone marrow-derived mononuclear cells for therapeutic neovascularization in acute ischemia. Arterioscler Thromb Vasc Biol.

[21] **Guidance for FDA Reviewers and Sponsors: Content and Review of Chemistry, Manufacturing, and Control (CMC) Information for Human Gene Therapy Investigational New Drug Applications (INDs) US Dept of health, FDA, CBER”.** 2008.

[22] **FDA/CBER Guidance for Industry “Bioanalytical Method Validation”.** 2001.

[23] **ICH Q2 (R1) “Validation of Analytical Procedures: Text and Methodology”.** 2005.

[24] Alsousou J, Ali A, Willett K, Harrison P (2013). The role of platelet-rich plasma in tissue regeneration. Platelets.

[25] Assmus B, Tonn T, Seeger FH, Yoon CH, Leistner D, Klotsche J, Schachinger V, Seifried E, Zeiher AM, Dimmeler S (2010). Red blood cell contamination of the final cell product impairs the efficacy of autologous bone marrow mononuclear cell therapy. J Am Coll Cardiol.

[26] Schachinger V, Tonn T, Dimmeler S, Zeiher AM (2006). Bone-marrow-derived progenitor cell therapy in need of proof of concept: design of the REPAIR-AMI trial. Nat Clin Pract Cardiovasc Med.

[27] Grisendi G, Anneren C, Cafarelli L, Sternieri R, Veronesi E, Cervo GL, Luminari S, Maur M, Frassoldati A, Palazzi G, Otsuru S, Bambi F, Paolucci P, Pierfranco C, Horwitz E, Dominici M (2010). GMP-manufactured density gradient media for optimized mesenchymal stromal/stem cell isolation and expansion. Cytotherapy.

[28] Delewi R, Hirsch A, Tijssen JG, Schachinger V, Wojakowski W, Roncalli J, Aakhus S, Erbs S, Assmus B, Tendera M, Goekmen Turan R, Corti R, Henry T, Lemarchand P, Lunde K, Cao F, Huikuri HV, Sürder D, Simari RD, Janssens S, Wollert KC, Plewka M, Grajek S, Traverse JH, Zijlstra F, Piek JJ (2014). Impact of intracoronary bone marrow cell therapy on left ventricular function in the setting of ST-segment elevation myocardial infarction: a collaborative meta-analysis. Eur Heart J.

[29] Lasala GP, Minguell JJ (2011). Vascular disease and stem cell therapies. Br Med Bull.

[30] Perin EC, Willerson JT, Pepine CJ, Henry TD, Ellis SG, Zhao DX, Silva GV, Lai D, Thomas JD, Kronenberg MW, Martin AD, Anderson RD, Traverse JH, Penn MS, Anwaruddin S, Hatzopoulos AK, Gee AP, Taylor DA, Cogle CR, Smith D, Westbrook L, Chen J, Handberg E, Olson RE, Geither C, Bowman S, Francescon J, Baraniuk S, Piller LB, Simpson LM (2012). Effect of transendocardial delivery of autologous bone marrow mononuclear cells on functional capacity, left ventricular function, and perfusion in chronic heart failure: the FOCUS-CCTRN trial. JAMA.

[31] Gee AP, Richman S, Durett A, McKenna D, Traverse J, Henry T, Fisk D, Pepine C, Bloom J, Willerson J, Prater K, Zhao D, Koç JR, Ellis S, Taylor D, Cogle C, Moyé L, Simari R, Skarlatos S (2010). Multicenter cell processing for cardiovascular regenerative medicine applications: the Cardiovascular Cell Therapy Research Network (CCTRN) experience. Cytotherapy.

[32] Grajek S, Popiel M, Gil L, Breborowicz P, Lesiak M, Czepczynski R, Sawinski K, Straburzynska-Migaj E, Araszkiewicz A, Czyz A, Kozłowska-Skrzypczak M, Komarnicki M (2010). Influence of bone marrow stem cells on left ventricle perfusion and ejection fraction in patients with acute myocardial infarction of anterior wall: randomized clinical trial: Impact of bone marrow stem cell intracoronary infusion on improvement of microcirculation. Eur Heart J.

[33] Huikuri HV, Kervinen K, Niemela M, Ylitalo K, Saily M, Koistinen P, Savolainen ER, Ukkonen H, Pietila M, Airaksinen JK, Knuuti J, Mäkikallio TH (2008). Effects of intracoronary injection of mononuclear bone marrow cells on left ventricular function, arrhythmia risk profile, and restenosis after thrombolytic therapy of acute myocardial infarction. Eur Heart J.

[34] Bartsch T, Brehm M, Zeus T, Kogler G, Wernet P, Strauer BE (2007). Transplantation of autologous mononuclear bone marrow stem cells in patients with peripheral arterial disease (the TAM-PAD study). Clin Res Cardiol.

[35] *European Pharmacopoeia 7.0, Section 5.1.10 (Guidelines for using the test for bacterial endotoxins).* Strasbourg, FR: European Directorate for the Quality of Medicines & HealthCare; 2010.

[36] Schachinger V, Assmus B, Britten MB, Honold J, Lehmann R, Teupe C, Abolmaali ND, Vogl TJ, Hofmann WK, Martin H, Dimmeler S, Zeiher AM (2004). Transplantation of progenitor cells and regeneration enhancement in acute myocardial infarction: final one-year results of the TOPCARE-AMI Trial. J Am Coll Cardiol.

[37] Lunde K, Solheim S, Aakhus S, Arnesen H, Abdelnoor M, Egeland T, Endresen K, Ilebekk A, Mangschau A, Fjeld JG, Smith HJ, Taraldsrud E, Grøgaard HK, Bjørnerheim R, Brekke M, Müller C, Hopp E, Ragnarsson A, Brinchmann JE, Forfang K (2006). Intracoronary injection of mononuclear bone marrow cells in acute myocardial infarction. N Engl J Med.

[38] Assmus B, Walter DH, Seeger FH, Leistner DM, Steiner J, Ziegler I, Lutz A, Khaled W, Klotsche J, Tonn T, Dimmeler S, Zeiher AM (2013). Effect of shock wave-facilitated intracoronary cell therapy on LVEF in patients with chronic heart failure: the CELLWAVE randomized clinical trial. JAMA.

[39] Walter DH, Krankenberg H, Balzer JO, Kalka C, Baumgartner I, Schluter M, Tonn T, Seeger F, Dimmeler S, Lindhoff-Last E, Zeiher AM (2011). Intraarterial administration of bone marrow mononuclear cells in patients with critical limb ischemia: a randomized-start, placebo-controlled pilot trial (PROVASA). Circ Cardiovasc Interv.

[40] Cao F, Sun D, Li C, Narsinh K, Zhao L, Li X, Feng X, Zhang J, Duan Y, Wang J, Liu D, Wang H (2009). Long-term myocardial functional improvement after autologous bone marrow mononuclear cells transplantation in patients with ST-segment elevation myocardial infarction: 4 years follow-up. Eur Heart J.

[41] Hirsch A, Nijveldt R, van der Vleuten PA, Tijssen JG, van der Giessen WJ, Tio RA, Waltenberger J, ten Berg JM, Doevendans PA, Aengevaeren WR, Zwaginga JJ, Biemond BJ, van Rossum AC, Piek JJ, Zijlstra F (2011). Intracoronary infusion of mononuclear cells from bone marrow or peripheral blood compared with standard therapy in patients after acute myocardial infarction treated by primary percutaneous coronary intervention: results of the randomized controlled HEBE trial. Eur Heart J.

[42] Janssens S, Dubois C, Bogaert J, Theunissen K, Deroose C, Desmet W, Kalantzi M, Herbots L, Sinnaeve P, Dens J, Maertens J, Rademakers F, Dymarkowski S, Gheysens O, Van Cleemput J, Bormans G, Nuyts J, Belmans A, Mortelmans L, Boogaerts M, Van de Werf F (2006). Autologous bone marrow-derived stem-cell transfer in patients with ST-segment elevation myocardial infarction: double-blind, randomised controlled trial. Lancet.

[43] Traverse JH, Henry TD, Pepine CJ, Willerson JT, Zhao DX, Ellis SG, Forder JR, Anderson RD, Hatzopoulos AK, Penn MS, Perin EC, Chambers J, Baran KW, Raveendran G, Lambert C, Lerman A, Simon DI, Vaughan DE, Lai D, Gee AP, Taylor DA, Cogle CR, Thomas JD, Olson RE, Bowman S, Francescon J, Geither C, Handberg E, Kappenman C, Westbrook L (2012). Effect of the use and timing of bone marrow mononuclear cell delivery on left ventricular function after acute myocardial infarction: the TIME randomized trial. JAMA.

[44] Ruiz-Salmeron R, de la Cuesta-Diaz A, Constantino-Bermejo M, Perez-Camacho I, Marcos-Sanchez F, Hmadcha A, Soria B (2011). Angiographic demonstration of neoangiogenesis after intra-arterial infusion of autologous bone marrow mononuclear cells in diabetic patients with critical limb ischemia. Cell Transplant.

[45] Peichev M, Naiyer AJ, Pereira D, Zhu Z, Lane WJ, Williams M, Oz MC, Hicklin DJ, Witte L, Moore MA, Rafii S (2000). Expression of VEGFR-2 and AC133 by circulating human CD34(+) cells identifies a population of functional endothelial precursors. Blood.

[46] Assmus B, Leistner DM, Schachinger V, Erbs S, Elsasser A, Haberbosch W, Hambrecht R, Sedding D, Yu J, Corti R, Mathey DG, Barth C, Mayer-Wehrstein C, Burck I, Sueselbeck T, Dill T, Hamm CW, Tonn T, Dimmeler S, Zeiher AM (2014). Long-term clinical outcome after intracoronary application of bone marrow-derived mononuclear cells for acute myocardial infarction: migratory capacity of administered cells determines event-free survival. Eur Heart J.

[47] Fadini GP, Losordo D, Dimmeler S (2012). Critical reevaluation of endothelial progenitor cell phenotypes for therapeutic and diagnostic use. Circ Res.

[48] Hill JM, Zalos G, Halcox JP, Schenke WH, Waclawiw MA, Quyyumi AA, Finkel T (2003). Circulating endothelial progenitor cells, vascular function, and cardiovascular risk. N Engl J Med.

[49] Seeger FH, Tonn T, Krzossok N, Zeiher AM, Dimmeler S (2007). Cell isolation procedures matter: a comparison of different isolation protocols of bone marrow mononuclear cells used for cell therapy in patients with acute myocardial infarction. Eur Heart J.

[50] Dawn B, Bolli R (2007). Bone marrow for cardiac repair: the importance of characterizing the phenotype and function of injected cells. Eur Heart J.

[51] Yang ZDSS, Kalka C (2010). Current developments in the use of stem cell for therapeutic neovascularisation: is the future therapy “cell-free”?. Swiss Med Wkly.

[52] Bravery CA, Carmen J, Fong T, Oprea W, Hoogendoorn KH, Woda J, Burger SR, Rowley JA, Bonyhadi ML, Van’t Hof W (2013). Potency assay development for cellular therapy products: an ISCT review of the requirements and experiences in the industry. Cytotherapy.

[53] **ICH Q1A(R2) “Stability Testing of New Drug Substances and Products”.** 2003.

[54] **ICH Q1E “Evaluation for Stability Data”.** 2003.

[55] **EudraLex - Volume 4 - Good manufacturing practice (GMP) Guidelines.** available at http://ec.europa.eu/health/documents/eudralex/vol-4.

